# COVID-19 Symptoms and Deaths among Healthcare Workers, United States

**DOI:** 10.3201/eid2808.212200

**Published:** 2022-08

**Authors:** Shao Lin, Xinlei Deng, Ian Ryan, Kai Zhang, Wangjian Zhang, Ese Oghaghare, DeeDee Bennett Gayle, Benjamin Shaw

**Affiliations:** University of Albany School of Public Health, Rensselaer, New York, USA (S. Lin, X. Deng, I. Ryan, K. Zhang, E. Oghaghare, B. Shaw);; Sun Yat-Sen University School of Public Health, Guangzhou, China (W. Zhang);; University at Albany College of Emergency Preparedness, Homeland Security and Cybersecurity, Albany, New York, USA (D. Bennett Gayle);; University of Illinois Chicago School of Public Health, Chicago, Illinois, USA (B. Shaw)

**Keywords:** COVID-19, coronavirus disease, SARS-CoV-2, severe acute respiratory syndrome coronavirus 2, viruses, respiratory infections, zoonoses, deaths, healthcare workers, demographics, health status indicators, United States

## Abstract

We evaluated whether demographics and COVID-19 symptoms predicted COVID-19 deaths among healthcare workers (HCWs) in the United States by comparing COVID-19 deaths in HCWs with 3 control groups (HCW nondeaths, non-HCW deaths, and non-HCW nondeaths) using a case–control design. We obtained patient-level data of 33 variables reported during January 1, 2020–October 12, 2021, in all US states. We used logistic regression analysis while controlling for confounders. We found that persons who were >50 years of age, male, Black, or Asian experienced significantly more deaths than matched controls. In addition, HCWs who died had higher risks for the most severe clinical indicators. We also found that the most indicative symptoms were preexisting medical conditions, shortness of breath, fever, cough, and gastrointestinal symptoms. In summary, minority, male, and older HCWs had greater risk for COVID-19 death. Severe clinical indicators and specific symptoms may predict COVID-19–related deaths among HCWs.

COVID-19 is one of the longest-lasting and largest global pandemics in history (*1*), but its typical symptoms and relevant clinical predictors are still unknown. By March 2022, >79 million Americans had contracted COVID-19, and >963,000 had died (*2*,*3*). Multiple studies have found that older adults (*4*,*5*) and persons with chronic medical conditions, such as diabetes, hypertension, and renal failure, were particularly susceptible to contracting COVID-19 (*6*,*7*).

Healthcare workers (HCWs) are another highly susceptible subpopulation (*8*–*10*) because of their time spent caring for COVID-19 patients (*11*). Of importance, 40% of HCWs identify as a racial minority; of those, 16% are Black, 13% Hispanic, and 7% Asian/other (*12*–*14*). Kirby reported that doctors from racial and ethnic minority communities were twice as likely to deal with patients without access to personal protective equipment (PPE) than White colleagues (*15*). Available data suggest that Black persons are more likely to hold jobs considered essential (e.g., HCW, medical assistant, food preparation, home care aide) than their White counterparts. In addition, ethnic minorities work disproportionately in the top 9 occupations exposed to COVID-19 and, therefore, are at high risk for infection (*16*). However, they are less likely to publicly express their workplace safety concerns for fear of job loss (*17*).

The initial surge in COVID-19 cases led to a profound increase in HCWs’ exposure to the virus. However, the extent to which increased exposure in HCWs led to increased risk for death—and which demographic characteristics, severity indicators, and symptoms best predict this risk—remains unclear. Most previous research has used non-HCWs as controls, leading to biases due to differences in occupation, education, and treatment accessibility. In addition, a nationwide study evaluating COVID-19 symptoms and deaths among HCWs is lacking, especially one that accounts for the second and third COVID-19 surges.

To fill these knowledge gaps, we used COVID-19 surveillance data from the Centers for Disease Control and Prevention (CDC) to compare the differences in demographic characteristics and symptoms between HCWs who died and those who did not (HCW deaths and nondeaths) and compared to the general population (non-HCW deaths and nondeaths). We also examined the temporal trends of COVID-19 infection and deaths in HCWs versus the general population.

## Methods

### Study Design and Controls

Our study population included all COVID-19 infection cases reported by the CDC. We used a case–control design to compare demographics and symptoms between HCW deaths and HCW nondeaths (control 1). HCW nondeaths were the primary control group, representing the source population and controlling for important confounders, including occupation, education, medical knowledge, and access to medical care. To compare our findings with previous research, we added 2 other reference groups from the US general population: non-HCW deaths (control 2), which is commonly used by other studies, and non-HCW nondeaths (control 3).

### Data Acquisition

We obtained data on laboratory-confirmed COVID-19 cases, probable cases, and deaths across the United States from the Restricted Access Dataset operated by the CDC. In January 2020, COVID-19 data collection commenced, and COVID-19 was added to the nationally notifiable condition list; on April 5, 2020, COVID-19 was classified as immediately notifiable, urgent (within 24 hours) (interim-20-ID-01). All states and territories were encouraged to enact laws in their jurisdictions to submit case notifications to CDC. CDC also requested that public health departments report all COVID-19 cases using standardized case report forms and case definitions for laboratory-confirmed or probable cases. This surveillance system includes patient-level data reported by all US territories and states. This study covers the timeframe January 1, 2020–October 12, 2021.

We obtained demographic and medical information for each record in this dataset, including COVID-19 case status (confirmed or probable case), date of first positive specimen collection, and demographics (sex, age group, race, ethnicity, and county and state of residence) (Table 1). We also obtained information on presence of severe COVID-19 clinical indicators and of less severe symptoms (Table 2). CDC suppressed data cells reporting <5 records and uncommon combinations of demographic characteristics (recoded to NA) to prevent releasing personally identifiable data.

**Table 1:** Comparison of sociodemographics among HCWs who died from COVID-19 versus 3 control groups, United States, January 1, 2020–October 12, 2021*

Variable	HCW deaths, no. (%)	Control 1: HCW nondeaths			Control 2: non-HCW deaths			Control 3: non-HCW nondeaths	
		No. (%)	p value†		No. (%)	p value‡		No. (%)	p value§
Sex									
F	893 (60.79)	356,553 (81.36)	<0.001		50,104 (44.97)	<0.001		2,240,363 (51.07)	<0.001
M	576 (39.21)	81,700 (18.64)			61,306 (55.03)			2,146,333 (48.93)	
Age group, y									
10–19	1 (0.07)	10,866 (2.48)	<0.001		104 (0.09)	<0.001		610,578 (13.89)	<0.001
20–29	27 (1.84)	109,220 (24.90)			536 (0.48)			794,483 (18.08)	
30–39	86 (5.85)	111,437 (25.41)			1,471 (1.32)			698,099 (15.89)	
40–49	160 (10.89)	90,003 (20.52)			3,613 (3.24)			633,574 (14.42)	
50–59	412 (28.05)	75,625 (17.24)			9,013 (8.08)			593,951 (13.52)	
60–69	551 (37.51)	36,657 (8.36)			18,621 (16.69)			423,500 (9.64)	
70–79	176 (11.98)	3,382 (0.77)			28,157 (25.23)			224,251 (5.10)	
80+	56 (3.81)	294 (0.07)			50,026 (44.83)			123,222 (2.80)	
Ethnicity									
Non-Hispanic	1,104 (80.29)	299,362 (80.63)	0.774		79,520 (76.84)	<0.001		2,588,535 (69.11)	<0.001
Hispanic	271 (19.71)	71,899 (19.37)			23,973 (23.16)			1,157,127 (30.89)	
Race									
White	530 (48.01)	208,585 (69.68)	<0.001		59,586 (74.93)	<0.001		1,878,386 (72.57)	<0.001
Black	300 (27.17)	46,883 (15.66)			10,644 (13.39)			350,133 (13.53)	
Asian	237 (21.47)	19,156 (6.40)			6,031 (7.58)			113,042 (4.37)	
American Indian/Alaska Native	0	497 (0.17)			292 (0.37)			13,813 (0.53)	
Native Hawaiian/other Pacific Islander	3 (0.27)	988 (0.33)			327 (0.41)			11,837 (0.46)	
Multiple/Other	34 (3.08)	23,253 (7.77)			2,640 (3.32)			221,324 (8.55)	
Month									
January	89 (7.00)	47,353 (11.52)	<0.001		11,209 (12.81)	<0.001		512,018 (12.61)	<0.001
February	30 (2.36)	15,997 (3.89)			3,455 (3.95)			217,736 (5.36)	
March	51 (4.01)	17,412 (4.24)			3,500 (4.00)			198,717 (4.90)	
April	95 (7.47)	35,111 (8.55)			7,079 (8.09)			268,465 (6.61)	
May	86 (6.77)	27,006 (6.57)			5,938 (6.79)			223,827 (5.51)	
June	347 (27.30)	43,318 (10.54)			10,658 (12.18)			318,614 (7.85)	
July	87 (6.85)	30,810 (7.50)			6,860 (7.84)			331,432 (8.16)	
August	107 (8.42)	34,256 (8.34)			7,088 (8.10)			405,229 (9.98)	
September	82 (6.45)	29,824 (7.26)			5,726 (6.54)			348,613 (8.59)	
October	49 (3.86)	23,615 (5.75)			4,492 (5.13)			238,337 (5.87)	
November	109 (8.58)	51,872 (12.62)			9,349 (10.68)			480,294 (11.83)	
December	139 (10.94)	54,316 (13.22)			12,149 (13.88)			516,249 (12.72)	
Season									
Spring	232 (18.25)	79,529 (19.36)	<0.001		16,517 (18.88)	<0.001		691,009 (17.02)	<0.001
Summer	541 (42.56)	108,384 (26.38)			24,606 (28.12)			1,055,275 (25.99)	
Fall	240 (18.88)	105,311 (25.63)			19,567 (22.36)			1,067,244 (26.29)	
Winter	258 (20.30)	117,666 (28.64)			26,813 (30.64)			1,246,003 (30.69)	

*A total of 440,044 healthcare workers were reported to have COVID-19, including 1,469 in the HCW deaths group and 438,575 in HCW nondeaths group. HCW, healthcare worker. †Comparison of HCW deaths vs. HCW nondeaths (control 1). ‡Comparison of HCW deaths vs. non-HCW deaths (control 2). §Comparison of HCW deaths vs. non-HCW nondeaths (control 3).

**Table 2:** Multivariable analyses for severe clinical indicators and reported symptoms among HCWs who died of COVID-19 compared with HCW nondeaths, United States, January 1, 2020–October 12, 2021*

Variables	HCW deaths, no. (%)	Control 1: HCW nondeaths	
		No. (%)	OR (95% CI)†
Total numbers	1,469	438,575	
Hospitalized	1,152 (83.84)	18,018 (5.20)	56.22 (47.40–66.70)
Admitted to ICU	663 (77.00)	1,530 (1.51)	102.51 (83.53–125.82)
Had pneumonia	281 (64.75)	4,363 (3.13)	30.97 (24.53–39.10)
Abnormal radiograph	207 (69.93)	2,647 (3.73)	28.75 (21.51–38.41)
Acute respiratory disease symptoms	138 (46.62)	1,120 (0.83)	61.96 (46.60–82.38)
MV intubation	296 (58.04)	306 (0.29)	230.94 (178.10–299.45)
Fever	305 (66.02)	57,293 (36.50)	3.11 (2.50–3.86)
Subjective fever	169 (52.98)	57,236 (37.68)	1.75 (1.37–2.23)
Chills	217 (48.98)	73,019 (42.77)	1.10 (0.90–1.35)
Myalgia	245 (53.38)	106,971 (59.10)	0.88 (0.72–1.08)
Running nose	69 (30.67)	63,003 (51.99)	0.51 (0.37–0.71)
Sore throat	106 (25.00)	72,053 (41.11)	0.65 (0.51–0.83)
Cough	440 (78.15)	124,345 (66.30)	1.65 (1.33–2.06)
Dyspnea, shortness of breath	399 (70.74)	51,547 (30.04)	6.06 (4.95–7.41)
Nausea/vomiting	127 (27.73)	39,179 (23.04)	1.49 (1.19–1.87)
Headache	195 (43.82)	121,960 (66.47)	0.50 (0.41–0.62)
Abdominal pain	47 (12.95)	17,180 (12.49)	1.03 (0.73–1.45)
Diarrhea	171 (37.58)	48,559 (28.68)	1.47 (1.20–1.82)
Underlying medical conditions	627 (88.31)	93,519 (43.94)	6.44 (4.95–8.39)

*The ORs in this table controlled for sex, age group, race, ethnicity, dominant periods of SARS-CoV-2 variants, and vaccination starting time. HCW, healthcare worker; ICU, intensive care unit; MV, mechanical ventilation; OR, odds ratio. †Comparison of HCW deaths vs. HCW nondeaths (control 1).

### Outcomes and Predictors

The health outcomes in this study were COVID-19–related deaths. Among HCW deaths and control groups 1, 2, and 3, a total of 97.8% were confirmed COVID-19 cases, and 2.2% were probable cases. We calculated fatality as the number of COVID-19 deaths divided by all COVID-19 cases in the United States. We used 20 predictors in the analysis, including demographic variables, severe COVID-19 clinical indicators, and less severe reported symptoms.

### Statistical Analysis and Confounders

We first compared all 20 predictor variables between HCW deaths and the 3 control groups using χ^2^ tests. We then developed logistic regression models by regressing fatality against each symptom predictor while controlling for potential confounders, including sex, age group, race, ethnicity, and periods of different SARS-CoV-2 variants and COVID-19 vaccines. We selected these confounders because they were associated with SARS-CoV-2 infection and various symptoms based on the literature and our data. We defined viral variant periods when specific SARS-CoV-2 variants were dominant in the United States (*18*): the original variants were dominant until March 20, 2021; the Alpha variant during March 21–May 30, 2021; the Delta variant during May 31–December 10, 2021; and Omicron since December 11, 2021. However, Omicron was not included because its dominance fell outside our study period (January 1, 2020–October 12, 2021). In addition, the first vaccine was given in America on December 14, 2020 (*19*). To account for these confounders, we included 3 dummy variables representing the periods of different SARS-CoV-2 variants and when vaccinations started in the United States and controlled these variables in each symptom model. Finally, we examined and compared the temporal trends of confirmed cases and deaths among HCWs and the general population. To reduce the instance of false-positive findings due to multiple testing, we conducted sensitivity analyses using the Bonferroni test method (Tables 1–3). We accomplished all data cleaning, analysis, and results using R version 3.6.1 (https://www.r-project.org).

**Table 3:** Multivariable analyses for severe clinical indicators and reported symptoms among HCWs who died of COVID-19 compared with non-HCW deaths and non-HCW nondeaths, United States, January 1, 2020–October 12, 2021*

Variables	HCW deaths, no. (%)	Control 2: non-HCW deaths			Control 3: non-HCW nondeaths	
		No. (%)	OR (95% CI)†		No. (%)	OR (95% CI)‡
Total numbers	1,469	1,436,898			4,394,371	
Hospitalized	1,152 (83.84)	81,094 (79.22)	1.24 (1.04–1.46)		239,761 (6.21)	46.16 (38.88–54.79)
Admitted ICU	663 (77.00)	33,066 (64.85)	1.57 (1.30–1.90)		25,754 (2.61)	76.80 (63.22–93.30)
Had pneumonia	281 (64.75)	13,722 (56.88)	1.32 (1.05–1.65)		53,506 (3.80)	26.55 (21.09–33.44)
Abnormal radiograph	207 (69.93)	9,997 (62.37)	1.19 (0.89–1.58)		34,944 (5.38)	21.89 (16.41–29.19)
Acute respiratory disease symptoms	138 (46.62)	7,994 (35.33)	1.46 (1.12–1.90)		11,452 (0.84)	62.43 (47.63–81.83)
MV intubation	296 (58.04)	8,729 (34.77)	1.85 (1.50–2.28)		4,123 (0.44)	156.19 (125.65–194.16)
Fever	305 (66.02)	15,800 (54.89)	1.39 (1.12–1.74)		532,385 (36.13)	3.38 (2.72–4.19)
Subjective fever	169 (52.98)	8,205 (36.34)	1.82 (1.42–2.34)		548,940 (36.23)	1.99 (1.56–2.54)
Chills	217 (48.98)	7,963 (30.64)	1.84 (1.48–2.28)		643,555 (38.88)	1.28 (1.04–1.57)
Myalgia	245 (53.38)	10,051 (36.51)	1.88 (1.52–2.32)		913,780 (51.95)	1.08 (0.88–1.32)
Running nose	69 (30.67)	3,244 (20.37)	1.75 (1.26–2.42)		495,995 (47.70)	0.58 (0.42–0.79)
Sore throat	106 (25.00)	4,225 (16.22)	1.59 (1.23–2.06)		641,523 (37.43)	0.71 (0.56–0.91)
Cough	440 (78.15)	23,085 (66.81)	1.63 (1.31–2.04)		1,159,870 (62.77)	1.70 (1.37–2.11)
Dyspnea, shortness of breath	399 (70.74)	23,545 (66.50)	1.11 (0.91–1.37)		405,746 (24.21)	5.95 (4.88–7.26)
Nausea/vomiting	127 (27.73)	5,304 (18.73)	1.42 (1.13–1.80)		297,213 (18.27)	1.60 (1.28–2.01)
Headache	195 (43.82)	6,821 (25.71)	1.87 (1.49–2.33)		1,068,424 (59.68)	0.59 (0.48–0.72)
Abdominal pain	47 (12.95)	2,079 (9.93)	1.07 (0.75–1.52)		136,869 (11.24)	1.05 (0.74–1.47)
Diarrhea	171 (37.58)	7,219 (25.56)	1.62 (1.31–2.01)		430,488 (25.88)	1.45 (1.18–1.78)
Underlying medical conditions	627 (88.31)	42,515 (93.01)	0.65 (0.49–0.85)		790,854 (42.64)	6.29 (4.82–8.21)

*The ORs in this table controlled for sex, age group, race, ethnicity, dominant periods of SARS-CoV-2 variants, and vaccination starting time. HCW, healthcare worker; ICU, intensive care unit; MV, mechanical ventilation; OR, odds ratio. †Comparison of HCW deaths vs. non-HCW deaths (control 2). ‡Comparison of HCW deaths vs. non-HCW nondeaths (control 3).

## Results

Among 6,271,313 laboratory-confirmed COVID-19 cases reported during January 1, 2020–October 12, 2021, by CDC, 7.02% (440,044) were in HCWs. The fatality rate among HCWs was 0.33% versus 24.64% for non-HCWs. The percentages we report represent the proportion of each specific variable (numerator) among HCW deaths or 1 of the 3 control groups (denominator) (Table 1). A total of 1,469 HCW deaths were reported among 440,044 cases. The proportion of male HCWs was significantly higher in HCW deaths (39.21%) compared with HCW nondeaths (control 1: 18.64%), although it was still lower than for non-HCWs (55.03% for deaths [control 2] and 48.93% for nondeaths [control 3]). The percentage of persons in the 50–59-year age group among HCW death cases was 28.05% and in the 60–69-year group, 37.51%; these rates were higher than those from all 3 control groups (8.08%–17.24% in the 50–59-year age group and 8.36%–17.24% in the 60–69-year group). The percentage of Hispanic persons in the HCW deaths category (19.71%) was not significantly different from other reference groups except for control 3 (30.89%). Furthermore, the percentages of Black (27.17%) and Asian (21.47%) persons in the HCW deaths category were greater than those in all 3 control groups (Black, 13.39%–15.66%; Asian 4.37%–7.58%).

Of note, we found that COVID-19 deaths among HCWs increased from March to June. June and then July–August contained the most HCW COVID-19 deaths compared with all controls: 27.30% versus 7.86%–12.18% in June and 42.56% versus 25.99%–28.12% in summer.

We conducted multivariate analysis for HCW deaths compared with 3 reference groups (Tables 2, 3). We calculated odds ratios (ORs) by severity indicators and COVID-19 related symptoms after adjusting for sex, age group, race/ethnicity, dominant periods of SARS-CoV-2 variants, and vaccination start time. All 6 severity indicators for COVID-19 were consistently higher in HCW death cases than in the 3 control groups (OR 1.24–230.94). The highest ORs occurred for mechanical ventilation, followed by intensive care unit admission, acute respiratory disease symptoms, hospitalization, pneumonia, and abnormal chest radiograph. In addition, compared with control 1, HCWs deaths showed significantly increased ORs for multiple symptoms, including specific preexisting medical conditions (OR 6.44, 95% CI 4.95–8.39), followed by shortness of breath, fever, subjective fever, cough, nausea/vomiting, and diarrhea (ORs 1.47–6.06; p<0.05). Chills, myalgia, and abdominal pain in HCW deaths group were not significantly different from those in the control groups. However, sore throat, running nose, and headache were significantly lower in the HCW deaths group than in the control 1 and control 2 groups. Those results remained significant and of similar magnitudes (<5% changes) after Bonferroni test adjustment (Tables 1–3). However, 4 severity indicators among HCWs (hospitalization, pneumonia, abnormal chest radiograph, and acute respiratory disease symptoms) became statistically nonsignificant compared with control 2 after the Bonferroni correction.

We compared the temporal patterns of COVID-19 infections and deaths among HCWs with those in the general population (Figure). Three surges of COVID-19 infections and deaths occurred in the United States around April 2020, July 2020, and November 2020–January 2021. Although infections peaked during November 2020–January in the general population, the highest death numbers occurred in the first surge (April 2020). Of note, the temporal trend of COVID-19 infections among HCWs was similar to that among the US general population. However, the COVID-19 deaths among HCWs declined after April 2020 and remained flat, whereas 2 subsequent death surges occurred among the general population.

**Figure:**
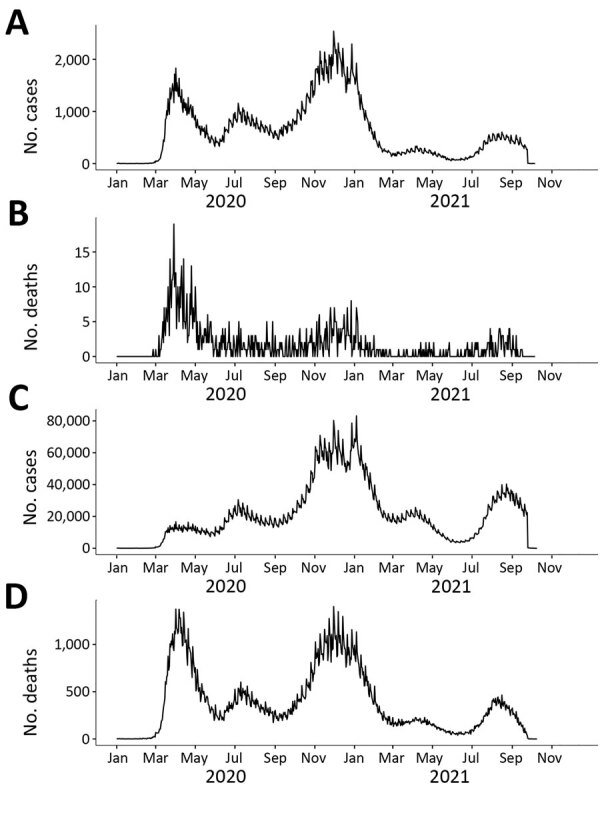
Comparison of COVID-19 cases and deaths among HCWs and in the non-HCW population, United States, January 2020–October 2021. A) Confirmed cases in HCW. B) HCW deaths. C) Confirmed cases in the non-HCW population. D) Confirmed deaths in non-HCW population. HCW, healthcare worker.

## Discussion

We found that HCWs who died of COVID-19 in the United States were disproportionately older (>50 years of age), male, and Black or Asian. Consistent with our findings, previous research found that older age groups are more vulnerable to COVID-19 infection and death, likely because of their lower immunity against viral infections and multiple preexisting medical conditions, both known to exacerbate COVID-19–related deaths (*5*,*20*,*21*). Of interest, we found that the highest risk for death among HCWs occurs in a relatively younger group (50–59 years of age) than the general population of hospitalized patients (>65 years of age). A possible explanation is that HCWs are generally a younger working population compared with retirees in the general population who suffer a higher COVID-19 burden. Unfortunately, there was no available literature in this area to confirm our findings.

Although this study found that female deaths were higher among HCWs than non-HCWs, female deaths were significantly lower than for HCW-controls, implying no significant increase in deaths among female HCWs after controlling for occupation. This finding could be attributable to the confounding factor of HCWs composition; that is, women are more likely to work in healthcare occupations (*22*). Therefore, using non-HCWs as a control group may lead to biases due to occupational confounders. On the other hand, worldwide, men were more likely to be infected by COVID-19 and have severe symptoms than were women, which is consistent with our findings of a 2-fold increase in death risk for male HCWs compared with male HCW nondeaths. Furthermore, several studies suggest that sex differences in the susceptibility to COVID-19 may be because of differences in immune response (*23*,*24*). These studies found higher plasma levels of innate immune cytokines, such as interleukin 8 and 18, among male patients but more robust T-cell activation among female patients infected by COVID-19.

Our findings regarding the increased risk for COVID-19 death among ethnic minority populations (Black and Asian) agree with several studies on the general population. For instance, Rogers et al. (*12*) and Kirby (*15*) reported an increased risk for COVID-19 deaths among non-Hispanic Black and Hispanic minority populations (*13*,*15*). In addition, two thirds of HCWs in the United Kingdom who died of COVID-19 identified as an ethnic minority (*25*). Another study found that COVID-19 infection and death were strongly linked with overcrowded neighborhoods, higher body mass index, and low incomes, all categories that are overrepresented in Hispanic and Black communities (*26*). However, we did not detect an increased risk for COVID-19 deaths among Hispanic HCWs, which could be caused by the small sample size (n = 271) or missing ethnicity information.

Our study also reported a significantly higher risk for COVID-19 deaths (3- to 5-fold) among Asian HCWs than in all 3 control groups. Sze et al. (*27*) reviewed 50 studies, including 18,728,893 COVID patients from the United States and the United Kingdom, and found that Black and Asian persons were at an increased risk for COVID-19 infection compared with White persons. Pooled adjusted OR for Black persons was 2.02 (95% CI 1.67–2.44), and for Asian persons, 1.50 (95% CI 1.24–1.83) (*27*). Nevertheless, few studies have reported that Asian persons are also at higher risk. Coronary heart disease, a high-risk comorbidity of COVID-19 death, is more common among Black persons, Asian persons, and persons of other ethnic minorities (*28*). Overcrowded households may be another risk factor associated with the spread of COVID-19 among Asian persons (*29*). Our results contradicted the belief we noted among Asian populations that they are at a lower risk of contracting COVID-19 because of genetic protection. Therefore, these findings are of critical public health importance in terms of communicating accurate COVID-19 information to particularly at-risk populations.

We found that almost one third of the HCW death cases in the United States occurred in June, and >40% occurred in the summer months (June–August) of 2020. The initial surge in COVID-19 cases led to a profound increase in HCWs’ exposure to the virus. This surge is likely the result of inadequate intensive care units and hospital beds, insufficient PPE supply, inadequate training and experience among HCWs, and heavy workloads due to a large and rapid influx of patients. Another study reported that HCWs with inadequate access to PPE had increased SARS-CoV-2 infection compared with those with adequate PPE access (*30*).

We found that the fatality rate in the US general population (2.48%) was more than 7-fold higher than that among HCWs (0.33%). This finding is consistent with that of Sahu et al. (*9*), who also found that the mortality rate among HCWs was 7 times lower than that among all cases (*10*). However, almost all severe indicators and symptoms were higher among HCW deaths than in the 3 control groups in this study, which may be because of HCWs’ proximity to and longer duration of exposure to COVID-19 patients. Alternatively, HCWs may have better access to healthcare and treatment, which prevented deaths in this population. Unfortunately, there is a paucity of literature regarding the severity indicators for COVID-19 to compare with our study.

We found that underlying conditions were the most important predictor of COVID-19 deaths among HCWs, followed by shortness of breath, fever, cough, nausea/vomiting, and diarrhea. Multiple studies found that chronic conditions were the most critical COVID-19 severity and death indicators in different countries (*6*,*7*). Other studies have shown that shortness of breath is another important symptom of COVID infection (*31*,*32*). Our findings that fever >100.4°F, even subjective fever (felt feverish), was consistently more common for HCW deaths than for the 3 control groups, indicating that fever may be an early indicator of disease severity. Another finding is that HCW death cases reported significantly higher gastrointestinal symptoms (diarrhea, nausea, vomiting, abdominal pain) than all 3 control groups. Consistent with our findings, Wiersinga et al. (*31*) reported that initial COVID-19 symptoms might include shortness of breath, fever, cough, nausea/vomiting, or diarrhea. The general public expects respiratory symptoms but may be unaware of gastrointestinal symptoms related to COVID-19. Therefore, this study may provide valuable insight for public education and severity prediction.

We also found that cough is among the top 3 reported symptoms of COVID-19 infections and deaths in HCWs and the general population (62.77%–78.15%). However, although headache (59.68%–66.47%) and myalgia (51.95%–59.10%) were the other 2 top symptoms among COVID-19 infection cases, preexisting conditions (88.31%–93.01%) and shortness of breath (66.5%–70%) were the commonly reported symptoms for COVID-19 deaths. In addition, we found that runny nose, sore throat, and headache symptoms were notably lower in HCW deaths than in nondeath controls, implying that these symptoms may not be essential predictors of COVID-19 death. Unfortunately, we found no relevant literature with which to compare our results.

Temporal patterns of infection and death in our study showed that, whereas COVID-19 deaths in the general US population experienced 3 distinct peaks, deaths among HCWs only peaked during the first surge. Deaths among HCWs went down after April 2020 and remained low. This finding is consistent with CDC reports and other studies that show a large initial surge and subsequent decline. The similar peaks in both HCWs and non-HCWs illustrate how quickly the COVID-19 pandemic spread from the general population to HCWs who took care of the deadliest cases (*2*,*22*,*33*). The flatter COVID-19 death trend after the first surge among HCWs could be attributed to their early and high immunization rate, improved PPE, access to healthcare facilities, and early detection and treatment for HCWs compared with the general population.

A strength of our study is that we used multiple reference groups to minimize different biases. We further validated our findings using HCW controls with similar exposure opportunities and socioeconomic backgrounds. In addition, using 2 general-population reference groups helped us examine how demographics and symptoms differed between HCWs and the general population. We also used dynamic, nationwide CDC surveillance data. These objective data reduce reporting bias, which is typically a major concern in studies relying on media reports or questionnaires. Finally, we controlled for several known risk factors for COVID-19, including demographics, different dominant SARS-CoV-2 variants, and the start of vaccinations.

Our findings illustrate how timely CDC surveillance data, reported every 2 weeks, can be used to monitor the temporal trend of infections and deaths among different populations. In addition, unique findings, such as HCW deaths increasing in younger age groups and in Asian persons, could be used in targeted interventions. Furthermore, clinical agencies could use the severe clinical indicators and symptoms we identified to predict deaths and plan hospital beds.

A limitation of our study is that, because the COVID-19 case surveillance system is passive, our data may underestimate the number of cases, although reporting cases to the CDC is federally mandated. In addition, the availability of diagnostic testing, resources, and the priorities of health officials may influence the completeness of reporting. To address this issue, we included total cases (both laboratory-confirmed and probable cases); most COVID-19 deaths (>99%) were laboratory confirmed. Although the case report form captures severity indicators, these data may be inaccurate or underreported because some outcomes were unknown at the time of reporting. We repeated the analyses multiple times using different lengths of the cohort after the initial analysis by adding updated data, and the results are robust. Missing values, especially demographic information for death cases in the general population, are also important limitations, suggesting that control 2 would not be a good control group. Furthermore, we examined race and ethnicity variables separately and could not combine these 2 variables because of a lack of personal identifiers and the arbitrary coding in the data; only 1 variable (race or ethnicity) was coded. We used race/ethnicity as a surrogate for sociodemographic status; however, household income and deprivation indices, which correlate with COVID infection, were not available. Although multiple testing may cause false positives, our results are robust after Bonferroni correction, a conservative test used to protect from type I error.

Another challenge we faced was determining whether a reported death was caused by COVID-19 or other diseases. We believe that COVID-19 was the primary cause of death for the cases we used because the death-related question from the CDC questionnaire is specific to COVID-19 (i.e., “Did the patient die as a result of this illness [COVID-19]?”). In addition, similar to other infectious diseases under mandatory reporting, all COVID-19 cases and causes of death were confirmed and validated by hospitals or health departments. Finally, we could not separate unique infections from reinfections because no personal identifier is available. However, this problem may not have a substantial effect for several reasons. We aimed to examine fatality (deaths per infection) rather than infection. Although death was almost certainly reported only once, infections could be reported multiple times because of reinfection. Therefore, we may have underestimated fatalities because reinfections increased the denominator. This underestimation bias is likely similar between the HCW deaths and HCW controls because of identical occupations and similar sociodemographic status, which would be nondifferential and toward the null. When comparing HCW deaths to non-HCW controls, the denominator, including reinfection numbers, is likely larger among HCWs than the general population because of HCWs’ frequency and duration of exposure to patients. Therefore, the fatality ratio of these 2 groups may have been underestimated. Finally, the effect of such bias may be minimal because most COVID-19 reinfections occurred when the Omicron variant was dominant (86.9%) after December 31, 2021 (*34*), which occurred after our study period (January 1, 2020–October 12, 2021). For instance, the COVID-19 reinfection rate in New York through December 31, 2021, was 0.56%, but as of January 1, 2022, it was 3.74% (*34*).

In conclusion we found that HCWs who were >50 years of age, male, Black, or Asian experienced higher deaths from COVID-19. In addition, HCW COVID-19 patients experienced fewer deaths but significantly higher risks for the most severe indicators than the 3 reference groups. We also found that underlying conditions, shortness of breath, fever, cough, nausea/vomiting, and diarrhea were the most relevant indicators for COVID-19–related deaths among HCWs. Conversely, runny nose, sore throat, and headache may not be critical indicators for COVID-19 death in this population.
